# Simulation-Aided Design of Tubular Polymeric Capsules for Self-Healing Concrete

**DOI:** 10.3390/ma10010010

**Published:** 2016-12-24

**Authors:** Branko Šavija, João Feiteira, Maria Araújo, Sutima Chatrabhuti, Jean-Marie Raquez, Kim Van Tittelboom, Elke Gruyaert, Nele De Belie, Erik Schlangen

**Affiliations:** 1 Microlab, Faculty of Civil Engineering and Geosciences, Delft University of Technology, Stevinweg 1, 2628CN Delft, The Netherlands; b.savija@tudelft.nl (B.S.); erik.schlangen@tudelft.nl (E.S.); 2 Magnel Laboratory for Concrete Research, Department of Structural Engineering, Faculty of Engineering and Architecture, Ghent University, Technologiepark Zwijnaarde 904, 9052 Ghent, Belgium; j.feiteira@ugent.be (J.F.); adelaide.araujo@ugent.be (M.A.); kim.vantittelboom@ugent.be (K.V.T.); elke.gruyaert@ugent.be (E.G.); 3 Polymer Chemistry and Biomaterials Group, Department of Organic and Macromolecular Chemistry, Faculty of Sciences, Ghent University, Krijgslaan 281, Building S4-bis, 9000 Ghent, Belgium; 4 Laboratory of Polymeric and Composite Materials (LPCM), Center of Innovation and Research in Materials and Polymers (CIRMAP), University of Mons, Place du Parc 23, B-7000 Mons, Belgium; sutima.chatrabhuti@umons.ac.be (S.C.); jean-marie.raquez@umons.ac.be (J.-M.R.)

**Keywords:** polymers, capsules, self-healing, concrete, cracks, simulation

## Abstract

Polymeric capsules can have an advantage over glass capsules used up to now as proof-of-concept carriers in self-healing concrete. They allow easier processing and afford the possibility to fine tune their mechanical properties. Out of the multiple requirements for capsules used in this context, the capability of rupturing when crossed by a crack in concrete of a typical size is one of the most relevant, as without it no healing agent is released into the crack. This study assessed the fitness of five types of polymeric capsules to fulfill this requirement by using a numerical model to screen the best performing ones and verifying their fitness with experimental methods. Capsules made of a specific type of poly(methyl methacrylate) (PMMA) were considered fit for the intended application, rupturing at average crack sizes of 69 and 128 μm, respectively for a wall thickness of ~0.3 and ~0.7 mm. Thicker walls were considered unfit, as they ruptured for crack sizes much higher than 100 μm. Other types of PMMA used and polylactic acid were equally unfit for the same reason. There was overall good fitting between model output and experimental results and an elongation at break of 1.5% is recommended regarding polymers for this application.

## 1. Introduction

An increasing amount of research on encapsulation of healing agents for self-healing concrete has been published during the last decade. The existing research is focusing mainly on the micro-encapsulation of liquid healing agents in spherical capsules with typical sizes in the range of a few micrometers up to a few millimeters, a technique borrowed from self-healing polymeric materials [1], but also on the encapsulation in larger, proof-of-concept tubular capsules with typical inner diameter of 3–4 mm. In the context of self-healing concrete, the healing agents commonly encapsulated are bacterial spores in a solution, liquid mineral compounds, or polymer precursors [2,3].

Encapsulation protects the healing agents from undesired or premature reactions and degradation, to guarantee their availability at the onset of damage in the host concrete matrix. The liquid agents are then released from the capsules typically due to mechanical triggers—i.e., once a certain damage level in concrete is achieved, after which a crack crosses the capsule and eventually causes its rupture. More complex chemical triggers can also be used, by taking advantage of the ingress of chloride ions in maritime environment [4] or the decrease of pH [5] in concrete once cracks are formed. Thus, instead of a mechanical rupture, the chemical triggers induce a progressive degradation of the capsule.

Other than being able to effectively release the healing agent after the onset of damage, capsules need to meet other more basic, but challenging requirements. They have to resist the mechanical stresses experienced during placing of concrete, in case of pre-placement of the capsules in the formwork, or during the mixing process, if added to fresh concrete during mixing. The material used for the capsules also has to be compatible with both the healing agent on the inside and the aggressive, high pH environment of the concrete matrix on the outside. Furthermore, the capsule’s wall has to have adequate barrier properties, with low permeability and diffusivity, to be able to retain its content but also to avoid any undesired chemical interaction between the healing agent and the concrete matrix. Finally, capsules have to rupture for very low imposed deformations, so that they release their content when crossed by a crack in concrete. Cracks are typically limited by reinforced concrete design codes to be no more than 300 µm wide, depending on the exposure conditions [6].

It is the latter requirement, the need for capsules to rupture under small crack openings in a concrete matrix, that this study wishes to address. Tubular capsules extruded from different polymers are experimentally tested in order to assess their fitness for this application, with computer simulations aiding the initial screening of polymers.

Although tubular glass capsules have been used mostly as a proof-of-concept, they are ideal in terms of barrier properties and rupture for very small deformations. While filling and sealing of glass capsules is definitely possible at a large scale, as they are already used in the pharmaceutical and adhesive industries, polymeric capsules are potentially easier to manufacture, due to their lower processing temperatures and the possibility for integrated extrusion, filling, and sealing steps. Despite this, the development of tubular, polymeric capsules for self-healing concrete has been seldom studied [7,8].

Moreover, the development and design of capsules for self-healing concrete can greatly benefit from numerical simulations. Numerical simulations are, in general, less costly than laboratory tests and can help in reducing the number of expensive experiments. So far, however, self-healing concrete has mostly been developed using trial-and-error procedures, with little optimization. There is thus a great need for robust numerical models. In Schlangen and Joseph [9], a review of modelling work related to self-healing concrete is given. To date, the majority of numerical models available in the literature deals with autogenous self-healing [10,11,12], i.e., by taking into account the innate ability of concrete to heal cracks by carbonation or continued hydration, for example.

Models used to design engineered self-healing concrete are less common. For example, Joseph [13] developed a numerical model able to describe the release of glue from a tubular system and the self-healing effect. On the other hand, several models have been proposed for assessing the probability of capsule breakage during fracture in concrete [14,15,16]. At present, these simple models may be used for selecting the appropriate dosage of the self-healing agent. A major drawback of these models is that they do not consider the mechanical properties of the capsule material and its interaction with the cementitious matrix. This interaction has a major impact on the crack propagation [17,18]. It is in this aspect that numerical models can be advanced. Other researchers still are focusing on the complex mechanism of bonding between capsule and matrix [19] and on the input necessary to develop models that simulate the release and dispersion of healing agent once the capsules are ruptured [20].

In this study, numerical modelling is used to simulate breakage of tubular capsules made of different polymers and with various wall thicknesses. This makes it possible to reduce the number of experiments and quickly reject materials that are not suitable, regarding their capability to rupture when crossed by cracks in concrete.

## 2. Experimental Testing

### 2.1. Extruded Hollow Tubes

In this study, five polymers have been selected as encapsulation materials. Polystyrene (PS) was supplied by BASF AG (Ludwigshafen, Germany; polystyrol VPT granule, M_n_ = 195,000 g/mol), polylactic acid (PLA) was obtained from NatureWorks (Blair, NE, USA; PLA 4032D, 1.4% D-isomer). Two types of poly(methyl methacrylate) were used. PMMA_1 was kindly supplied by Evonik Performance Materials (Darmstadt, Germany; Plexiglas 8909, M_n_ = 38,000 g/mol) and PMMA_2 was also obtained from Evonik Performance Materials (Plexiglas 8N, M_n_ = 50,000 g/mol). PMMA_1-PEG was obtained by melt-blending PMMA_1 with 20 wt % of polyethyleneglycol monomethylether (PEG), which was purchased from Fluka (Buchs, Germany; mPEG2000, M_n_ = 2000 g/mol).

For the melt-blending, PMMA_1 was dried overnight under vacuum in an oven at 60 °C before compounding in a Brabender (Duisburg, Germany) mixer at 210 °C and a speed of 30 rpm for 3 min. PEG was added after PMMA was completely molten and the compounding process proceeded at 70 rpm for 7 min.

PEG was added as a plasticizer for PMMA_1 to assess the feasibility of this option as a way to improve the chances of the capsules surviving the mixing process of concrete. The addition of PEG would increase the ductility of the capsules, making them less prone to be damaged by the stresses seen during concrete mixing. The PEG would then be partially and progressively leached out into the moist concrete, further lowering the ductility of the capsules so that they would rupture when crossed by small cracks in concrete. Resistance to mixing in concrete will be addressed in a separate publication on a realistic implementation of self-healing concrete based on tubular capsules added to concrete during mixing. The strategy of using plasticizer migration to achieve evolving brittleness had already been tried by Gruyaert et al. [8] on capsules made out of ethyl cellulose, but no clear conclusions regarding its efficacy could be drawn.

The mechanical properties of the polymers were then determined by tensile tests performed on dog bone-shaped samples (70 mm overall length and a straight section 40 mm long with a cross section of 1.5 mm × 5.0 mm) on a Zwick (Leominster, UK) universal tensile testing machine with a load cell of 1000 N. A preload of 0.5 N was used, the extension monitored was given by the separation between the grips and the stress–strain curves were obtained at a speed of 1 mm/min at room temperature. The results of the tensile tests are listed in Table 1.

This study also considered glass as an encapsulation material for comparison purposes. The mechanical properties of glass are listed in Table 2 and were taken from literature that investigated the use of glass capsules for self-healing concrete [21] and from their respective technical sheet (Hilgenberg borosilicate glass 3.3).

To extrude the hollow tubes from which capsules were cut, pellets of polymer were dried in a hot air oven at 60 °C for one day before extrusion. The extrusion was performed on a Brabender (Duisburg, Germany) extruder equipped with a single screw and a tubular die with outer and inner diameters of 10 mm and 8 mm, respectively. The processing temperature was 225–235 °C with a screw speed of 10 min^−1^, while the conveyor speed was adjusted to get approximately the desired external diameter and wall-thickness, which were 6 mm and 0.30 mm respectively. For PMMA_1, tubes with thicker walls were also tested. The average dimensions of the tubular sections from which the capsules were cut are listed in Table 3.

### 2.2. Cracking of Mortar Specimens with Embedded Capsules

**Figure 1:**
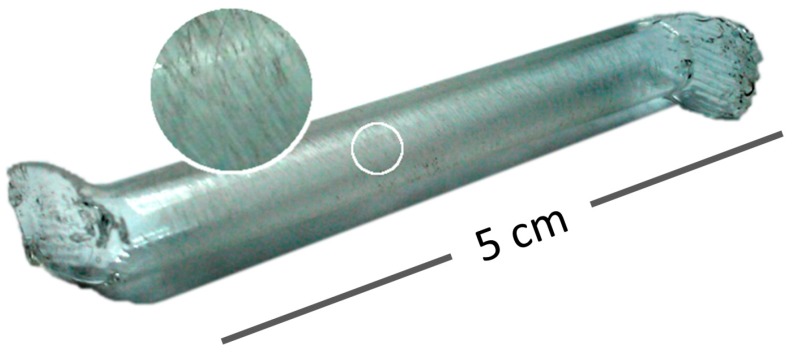
Capsule with molded hooked ends and sanded surface.

To create the capsules to be tested, sections 5 cm long were cut from the polymeric tubes. The ends of the capsules were heated and shaped to create hooks for improved mechanical locking once embedded in a mortar matrix. To additionally improve adhesion to the cementitious matrix, the capsules were sanded in a direction perpendicular to their length, as highlighted in Figure 1.

**Figure 2:**
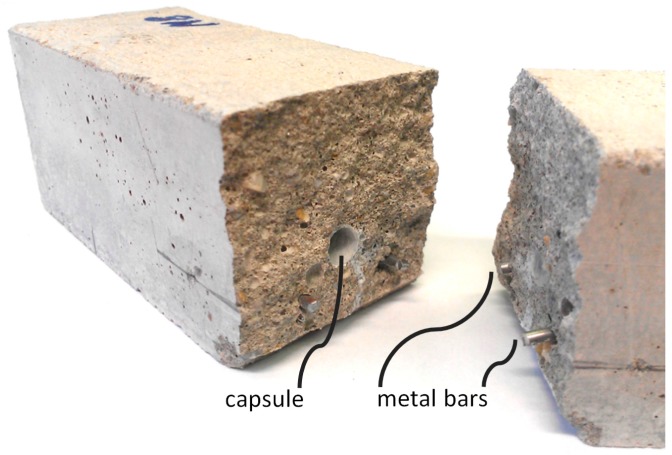
Split 4 cm × 4 cm × 16 cm specimen showing an embedded capsule that has been ruptured during bending.

The capsules were then embedded in reinforced mortar prisms with dimensions 4 cm × 4 cm × 16 cm by placing one capsule inside each mold, 1.3 cm from the bottom and centered relative to the sides, before pouring the mortar. The mortar mix consisted of CEM I 52.5 N, and had a sand-to-cement ratio of 3:1 and a water-to-cement ratio of 0.5. Mixing and molding were performed according to the EN 196-1 standard. The specimens contained also two reinforcing Ø2 mm smooth metal bars, placed 10 mm away from the sides and the bottom of the specimen, to avoid complete splitting during the cracking process. Figure 2 shows the relative positions of the capsule and the reinforcement bars on a specimen split in half.

**Figure 3:**
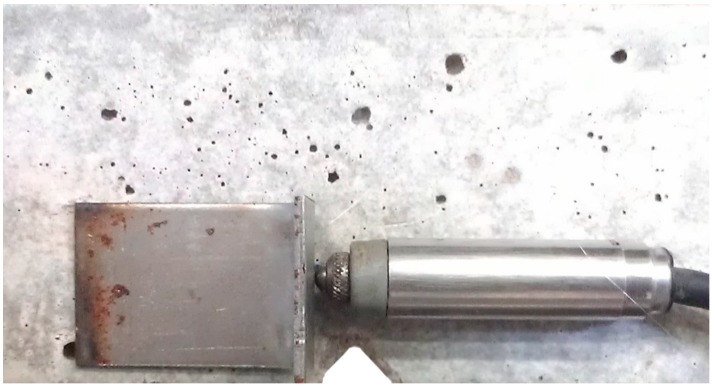
LVDT positioned at the side face of a specimen to measure the crack size at the height of the embedded capsule.

To create and progressively widen cracks that crossed the embedded capsules, the mortar prisms were loaded in a three-point bending test controlled by an external linear variable differential transformer (LVDT) (Solartron, Leicester, UK) with a 1 mm range. The LVDT was attached at one of the sides of the specimen, parallel to the embedded capsule and at the same height as its bottom fiber (Figure 3), so that it effectively measured the size of the crack crossing the capsule. To standardize the orientation of the crack, a triangular notch was molded into the bottom of the specimens, at half of their length.

## 3. Modelling Principle

In this work, the Delft lattice model is used to simulate rupture of tubular capsules subjected to mechanical loading. Lattice type models have been first used by theoretical physicists to model fracture mechanisms in heterogeneous materials [22]. This type of model has been adopted by various authors to simulate concrete fracture [23,24]. Fracture processes in other anisotropic or heterogeneous materials have been successfully simulated by lattice models as well—e.g., wood [25] or porous reactor core graphite [26].

**Figure 4:**
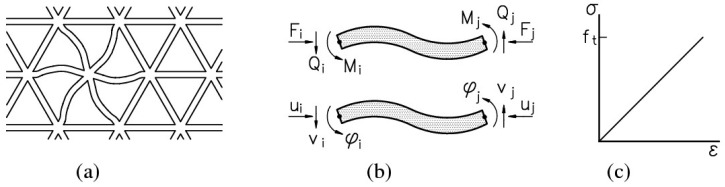
Lattice of beam elements (**a**); definition of forces and degrees of freedom (**b**); stress-strain relation of beam element (**c**).

In these models, material is discretized as a set of small truss or beam elements that can transfer forces (Figure 4). In the Delft lattice model as used herein, all individual elements exhibit linear elastic behavior. The fracture simulation is achieved by performing a linear elastic analysis of the lattice under loading, and removing an element which exceeds a prescribed fracture criterion (e.g., strength, strain, or energy) from the mesh. This analysis is then repeated in a step-wise manner, removing a single element in each-step. Thus, a non-linear analysis is performed by actually performing a number of linear analyses. Using this method, realistic crack patterns are found. Furthermore, even though individual elements all behave brittle, a ductile global response is achieved. Details about the underlying elastic equations as well as the full computational procedure of the model are available in [27,28].

In the present work, a fracture criterion based on the tensile stress in beams is adopted. Normal force (*N*) and bending moments (*M_x_*, *M_y_*) are both taken into account by the following general relation:

(1) $$\sigma =\alpha_{N}\frac{N}{A}+\alpha_{M}\frac{\max(M_{X},M_{Y})}{W}$$

where *A* is the beam cross-sectional area, *W* the cross-sectional moment of resistance, $\alpha_{N}$ and $\alpha_{M}$ are the normal force influence factor and the bending influence factor. Their values are adopted herein as 1.0 and 0.05, respectively.

**Figure 5:**
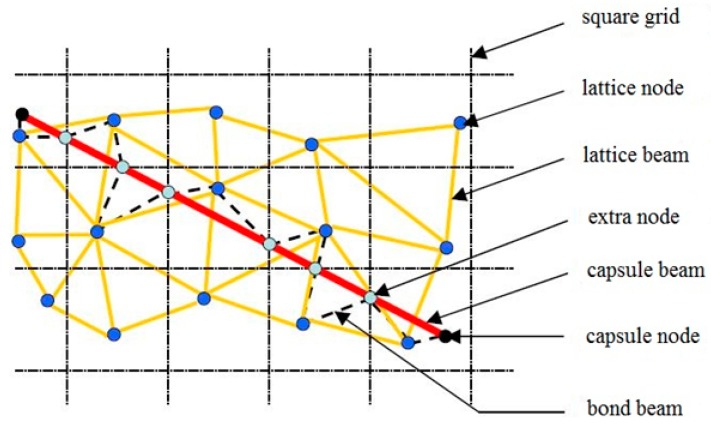
Schematic 2D representation of generation of capsule-lattice and their contact.

To simulate tubular carriers, a capsule is placed within the material domain. The capsule is connected to the matrix lattice elements through bond beam elements (Figure 5).

**Figure 6:**
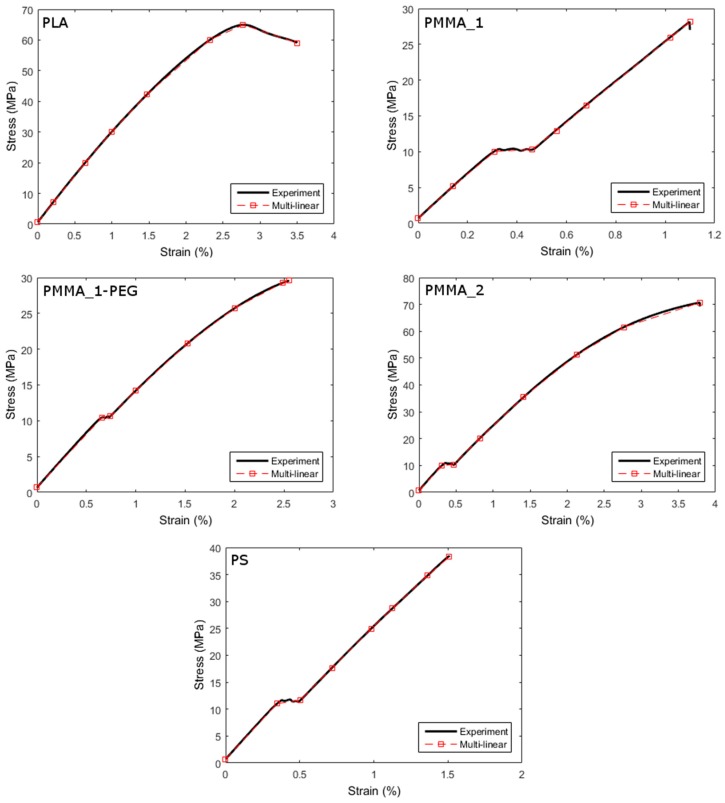
Experimental and schematized stress/strain relationships of encapsulation materials.

For matrix elements, a brittle fracture law is adopted, as presented in Figure 4. For the capsule elements, experimental data for each of the considered materials is input as a multi-linear stress/strain relation (Figure 6). Bond beams are not allowed to break in the present simulations, because a perfect bond is seen as a prerequisite for breakage of the tubular carriers.

**Figure 7:**
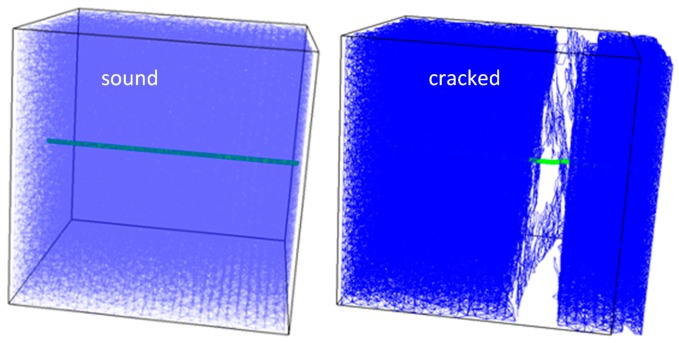
Lattice with an embedded tubular capsule.

In the simulations so far, a 30 mm × 30 mm × 30 mm mortar block with a single tubular capsule is simulated. It is schematically shown in Figure 7. It is subjected to uniaxial tension along the axis of the capsule, and the breakage of the capsule is monitored. The mortar is simulated as having a Young’s modulus of 20 GPa and a tensile strength of 3.5 MPa (which is within the range of values used in our previous work [29,30]).

## 4. Results

**Figure 8:**
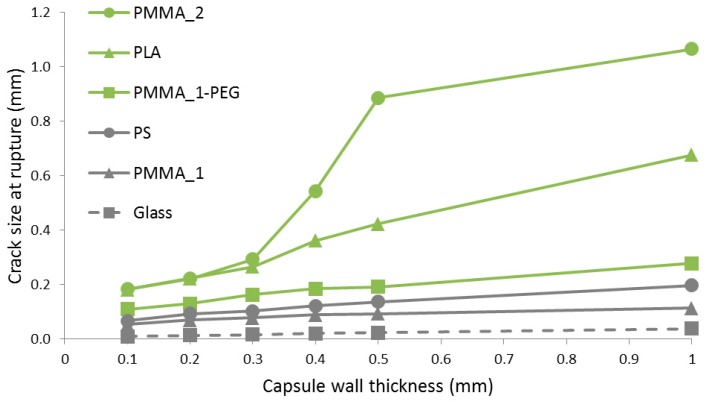
Model output for tubular capsules with an external diameter of 5 mm embedded in a mortar matrix under tensile stress.

The numerical model was used to simulate the mechanical response of capsules embedded in a cementitious matrix. This was performed for the different polymers listed in Table 1, considering capsules with a 5 mm external diameter and a wall thickness in the range 0.1–1.0 mm, to have a first assessment of their fitness. The dimensions used as input for the model at this stage are representative of the typical size of capsules that can be manufactured with the extrusion equipment used. As shown in Figure 8, only capsules made of polystyrene (PS) and a polymethyl methacrylate (PMMA_1) seem fit for the intended application, as they rupture for a crack size below the targeted limit of 100 µm (see introduction), however only if the wall thickness is kept under ~0.5 mm. Of the two materials, the crack size at rupture for capsules of PMMA_1 is the least affected by the wall thickness. It is also shown that capsules of both of these materials still rupture for a crack size several times higher than the size required to rupture glass capsules of similar dimensions. The glass curve was based on the properties listed in Table 2 and assumes a perfectly-elastic brittle behavior.

As a rule, the mechanical properties of polymers—such as tensile strength, stiffness, and ductility—rise with increasing molecular weight. This can be clearly observed in Figure 6, where PMMA_2 (M_n_ = 50,000) shows higher tensile strength and ductility than PMMA_1 (M_n_ = 38,000). As both types of PMMA have a similar Young’s modulus, the higher tensile strength (twice as high) and ductility of PMMA_2 result in larger elongation at break, which explains its much larger crack size at rupture. It can also be said that the inadequate performance of PLA capsules, which break only for crack sizes of ~0.2 mm or higher, is not necessarily intrinsic to the material type, depending also on the molecular weight of the PLA used.

Overall, different combinations of tensile strength, stiffness, and ductility of the different polymer types and molecular weights result in different values of elongation at break, which is the commanding factor for guaranteeing rupturing of capsules when crossed by small cracks in concrete. Given the good predicted performance of PMMA_1 and PS capsules shown in Figure 8, and according to their elongation at break listed in Table 1, an elongation of 1.5% or less is recommended for polymers considered as encapsulating materials in self-healing concrete. Polyethylene terephthalate (PET) and polyvinyl alcohol (PVA) could potentially also fulfill this requirement for low elongation at break. Although being water-soluble, PVA would not be the best solution for application in concrete. Other factors should also be considered, such as compatibility with the encapsulated healing agent and with the extrusion process. All polymers used in this study were equally easy to melt and flowed easily during extrusion, while their cost was not as relevant, as all selected polymers were inexpensive (<1 EUR/kg).

**Figure 9:**
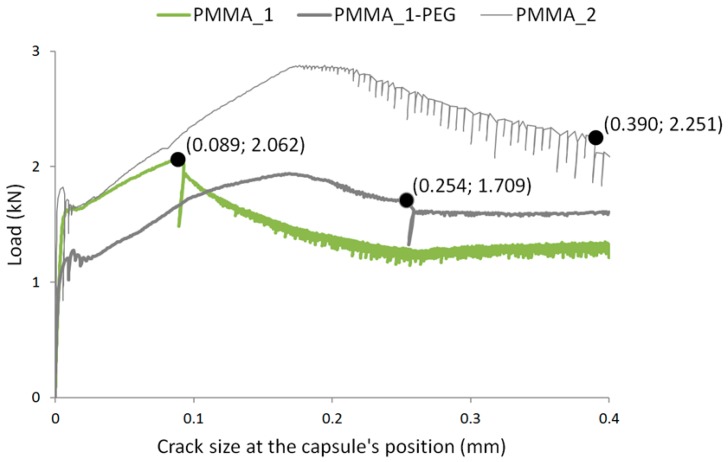
Characteristic load drops at the moment of rupturing of capsules embedded in mortar prims during three-point bending experimental tests. PMMA_1 curve is from the series with thinner walls of 0.31 mm.

The mechanical response of the capsules was then experimentally determined by embedding them in mortar specimens cracked under bending, where the capsules rupture under tensile stress once the crack crossing them reaches a critical size. Rupturing of the capsules induces a sharp load drop in the stress-displacement curves and a small reduction in the load capacity after that. These features were then used to experimentally determine the crack size at rupture of the capsules. Representative curves are shown in Figure 9 for the capsule types for which an average was possible to determine—i.e., for which all capsules ruptured before a 0.4 mm crack size was achieved.

**Figure 10:**
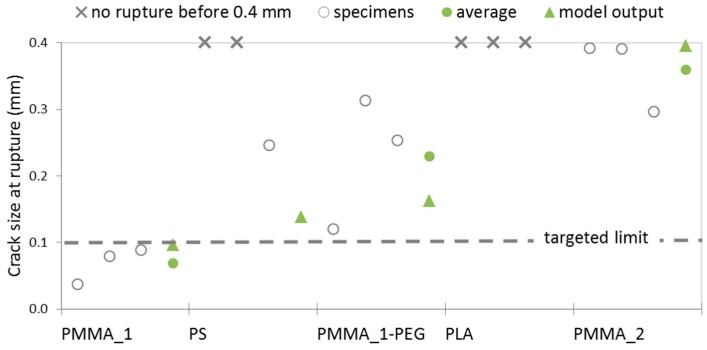
Crack size at the moment of rupturing of the capsules, in relation to the targeted maximum crack size of 100 µm.

The experimental data regarding the crack width at rupture for the different capsule types is plotted in Figure 10 and it confirms that the capsules extruded from PMMA_1 (wall thickness of 0.31 mm) rupture for a crack size below 100 µm, which makes them fit for the intended application. The results also show that PEG did not leach out of the PMMA_1-PEG material, or it did not leach out enough for capsules of this material to behave as capsules made out of PMMA_1, instead rupturing for crack sizes more than twice as large. The experimental data for PMMA_1-PEG also shows more scattering and the average crack size at rupture ended up being larger than the model output, when the opposite would be expected if there had been leaching of the PEG plasticizer. Accordingly, it was for these capsules that the model output showed its maximum deviation from the experimental results, at ~29%. The scattering of experimental data for this polymer can be due to insufficient blending of PMMA and PEG, resulting in a polymer matrix that is not homogeneous. Given the good modelled performance for extruded PS, it was unexpected that two out of three capsules of this polymer did not rupture during the test, which achieved a maximum crack size of 400 µm. A possible explanation for this could be a somewhat worse adhesion of the PS material to the cementitious matrix, which would cause the capsules to slip instead of deforming. An additional reason could be that the mechanical properties of PS were affected by the extrusion process, which would then result in a mismatch between the experimental results and the model, which used as input the mechanical properties prior to extrusion. Mechanical properties can also be affected by exposure to the alkaline environment of concrete, but that is unlikely to be the case, given the relatively short exposure of 14 days.

For the remaining polymers, the crack width at rupture was much higher than the targeted limit of 100 µm, as expected from the model output and confirming that these polymers are not good candidates for the intended application. For PMMA_2, the model output was very similar to the average crack size at rupture determined experimentally, with a deviation of ~10%. Regarding PLA capsules, it was not possible to assess how accurate the model was, given that their crack size at rupture is larger than the range of the experimental test and estimated by the numerical model to be 0.547 mm.

**Figure 11:**
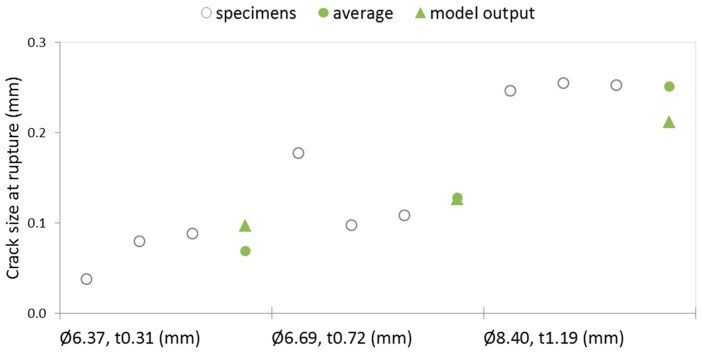
Crack size at the moment of rupturing of PMMA_1 capsules with different external diameter (Ø) and wall thickness (t), according to both the numerical model and the experimental results.

For the best performing PMMA_1 polymer, capsules with thicker walls were then extruded and experimentally tested and the results were compared to the model output based on the exact same dimensions. The results are plotted in Figure 11. The average crack size at rupture determined experimentally followed the same trend of the model output, i.e., an increase in crack size at rupture with increasing wall thickness (for similar external diameters), with a maximum deviation from the model of ~16% for the capsules with a 1.19 mm wall. The absolute values of crack width at rupture determined experimentally were 69 ± 22 µm, 128 ± 36 µm, and 251 ± 4 µm, respectively for capsules with an average wall thickness of 0.31, 0.72, and 1.19 mm.

Finally, to assess how the results were affected by the treatment of the capsules, which included sanding of the outer surface and molding hooks at the ends (Figure 1), tests were also performed on smooth capsules without the treatment. The capsules used were from the same batch as those identified in Figure 11 as having a wall thickness of 0.72 mm. Without the treatment, the capsules did not rupture within the testing range, which achieves a crack size of 400 µm. Reintroduction of only the sanding step, without the molded hooks at the ends, re-established rupturing for a similar range of crack sizes (111 ± 18 µm).

This proves also that it is unlikely that the poor performance of the PS capsules was related to a lack of adhesion, since the good adhesion between the best performing PMMA_1 capsules and the cement paste matrix was essentially mechanical in nature and achieved due to the roughness of the capsule’s surface introduced by sanding it, i.e., not due to a particularly good compatibility between PMMA and the cement paste.

## 5. Conclusions

The numerical model identified a wide range of performances for the polymeric capsules and was confirmed to be a useful tool for preliminary screening of materials to be used for encapsulation. The model’s output showed only two types of capsules (PMMA_1, PS) potentially rupturing for crack sizes below 100 µm, i.e., the maximum limit after which they should rupture and release the contents in the context of self-healing concrete. The low elongation at break of these polymers was a critical factor in achieving good performance and thus it was suggested that an elongation of less than 1.5% is recommended for this application.

For the PMMA_1 capsules, the experimental results agreed well with the model’s output, which showed a maximum deviation of 16%. Capsules with ~0.3 mm thick walls were considered fit for application in self-healing concrete, as they ruptured when crossed by an average crack size of 69 µm, while capsules with ~0.7 mm walls ruptured slightly above the targeted limit, for an average crack size of 128 µm. The latter size can potentially still be used for the considered application, but PMMA_1 capsules with ~1.2 mm were considered unfit. Regarding the PS capsules, the experimental results showed them to rupture only for very large crack sizes, unlike the result foreseen by the numerical model. The reason for this was not clear, although a lack of adhesion was improbable, as proper adhesion was proven to depend mainly on the abrasive treatment applied to the surface of all capsule types. Changes to this material during extrusion were thought to be the reason for the unexpected poor performance of PS capsules.

The experimental results confirmed that the remaining types of capsules (PMMA_1-PEG, PLA, PMMA_2) rupture for crack sizes much higher than the targeted limit and thus are unfit. The model output showed a high maximum deviation of 33% relative to average experimental results in the case of PMMA_1-PEG, most probably due to the fact that this material is a blend and thus less homogeneous than the other polymers, which resulted in higher scattering of results. Additionally, PEG was expected to leach out from the PMMA_1-PEG matrix towards the moist cementitious matrix, thus reducing the ductility of the capsules, but this was not confirmed. In the case of PMMA_2, its molecular weight was too high, making this material too ductile and strong compared to PMMA_1, but the model accurately predicted its performance, with a maximum deviation of 10%.

The presented modelling approach proved to be a valuable tool for designing capsules to be used as carriers of healing agents in self-healing concrete. The use of this and similar approaches in the future can provide guidance for experimental design and reduce the number of tests needed to design a robust self-healing system for use in concrete.

## Figures and Tables

**Table 1:** Mechanical properties of polymers determined on dog bone specimens.

Polymer	Tensile Strength (MPa)	Elongation at Break (%)	Young’s Modulus (MPa)
PLA	67.7 ± 0.5	4.3 ± 1.3	2946.6 ± 22.4
PMMA_1	29.1 ± 3.7	1.1 ± 0.1	2233.9 ± 16.0
PMMA_1-PEG	29.9 ± 0.3	2.8 ± 0.2	1299.7 ± 7.8
PMMA_2	61.5 ± 14.3	3.0 ± 1.0	2222.9 ± 30.6
PS	38.5 ± 1.8	1.5 ± 0.5	2254.5 ± 19.6

**Table 2:** Mechanical properties of glass according to literature [21].

Material	Tensile Strength (MPa)	Elongation at Break (%)	Young’s Modulus (MPa)
Glass	66	0.1	70,000

**Table 3:** Average dimensions of the capsules used for the experimental tests.

Polymer	External Diameter (mm)	Wall Thickness (mm)
PLA	7.42 ± 0.12	0.44 ± 0.11
PMMA_1	6.37 ± 0.25	0.31 ± 0.09
	6.69 ± 0.04	0.72 ± 0.02
	8.40 ± 0.08	1.19 ± 0.01
PMMA_1-PEG	6.34 ± 0.13	0.26 ± 0.07
PMMA_2	6.14 ± 0.09	0.26 ± 0.07
PS	6.44 ± 0.16	0.42 ± 0.13
